# Prostate specific antigen and relative prostate weight data on effect of *Tetracarpidium conophorum* leaf extract on testosterone-induced benign prostatic hyperplasia

**DOI:** 10.1016/j.dib.2018.06.104

**Published:** 2018-07-04

**Authors:** Omolola E. Omotosho, Bright P. Emmanuel, Oladipupo O. Ogunlade, Abiodun E. Salako

**Affiliations:** Department of Biochemistry, Covenant University, P.M.B. 1023, Km. 10, Idiroko road, Canaan land, Ota, Ogun State, Nigeria

## Abstract

Benign prostatic hyperplasia (BPH) is a common urological disorder of men, characterized by prostatic enlargement and urethral obstruction. In this study, BPH was induced in experimental groups by daily subcutaneous injections of testosterone propionate (TP) for 3 weeks. *Tetracarpidium conophorum* was administered daily by oral gavage at a dose of 100, 200 and 400 mg/kg BW of extract for three weeks, along with the TP injections and 5 mg/kg of finasteride for comparison. On day 21, the animals were sacrificed after anesthesia. Prostate were excised, weighed and used to determine relative prostate weight. Quantitative and qualitative phytochemical screening was also done and it showed the presence of flavonoids (0.370 mg/ml), tannins (0.458 mg/ml), phenols (0.508 mg/ml) and steroids (0.257 mg/ml). The prostate specific antigen level was evaluated, the result showed the data for extract group 200 mg/kg, 400 mg/kg, finasteride control group and BPH control group to be 0.186 ± 0.0023 ng/ml, 0.153 ± 0.005 ng/ml, 0.119 ± 0.0125 ng/ml and 0.332 ± 0.004 ng/ml respectively.

**Specifications Table**

**Table:** 

Subject area	Biochemistry
More specific subject area	Pharmacology and medicinal plant research
Type of data	Tables
How data was acquired	mean ± SEM, spectrophotometer, weighing balance, rotary evaporator, rat PSA ELISA kit.
Data format	Raw, Analyzed
Experimental factors	*Tetracarpidium conophorum* was macerated, soaked in 80% ethanol and extracted using rotary evaporator. Animals (Wistar rat) gotten were acclimatized, induced with benign prostatic hyperplasia and treated with *Tetracarpidium conophorum* and finasteride.
Experimental features	Quantitative and qualitative phytochemical screening was assayed on plant sample, prostate specific antigen and relative prostate weight were assayed on animal serum of different groups.
Data source location	Ota, Ogun State, Nigeria
Data accessibility	Data available within the article

**Value of the data**

• The data presented gives the quantitative assessment of certain phytochemicals responsible for the beneficial property of *Tetracarpidium conophorum* being used as an anti-hyperplastic agent.
• The data given may correlate with the results obtained using same or different plant in other regions.
• Data given can influence the development of a new drug against benign prostatic hyperplasia without extravagant side unlike finasteride.
• The data obtained from prostate specific antigen and relative prostate weight may correlate with data obtained in other regions with a different plant or similar plant.

## 1 Data

### 1.1 Qualitative phytochemical screening

See Table 1 here.

**Table 1:** Qualitative phytochemical screening in *Tetracarpidium conophorum.*

Phytochemicals	Present/Absent
Total phenol	Positive
Tannins	Positive
Flavonoid	Positive
Glycoside	Positive
Steroids	Positive
Saponin	Positive

### 1.2 Quantitative phytochemical screening

See Table 2 here.

**Table 2:** Quantitative phytochemicals data present in *Tetracarpidium conophorum*.

Phytochemical	Concentration (mg/ml)
Flavonoid	0.370
Total phenol	0.508
Tannin	0.458
Steroid	0.257

#### 1.2.1 Relative prostate weight determination in experimental animals

Relative prostate weight is used to evaluate the growth of benign prostatic hyperplasia in males (Table 3).

**Table 3:** Relative prostate weight (RPW) data in *Wistar* rats treated with *Tetracarpidium conophorum* and induced with benign prostatic hyperplasia.

**Parameters**	**RPW (g)**
Normal control	0.0009 ± 2.821^b^
BPH control	0.00015 ± 0.0001^a^
100 mg/kg *Tetracarpidium conophorum*	0.0013 ± 3.062^a^
200 mg/kg *Tetracarpidium conophorum*	0.0011 ± 0.0001^b^
400 mg/kg *Tetracarpidium conophorum*	0.0008 ± 0.0001^b^
Finasteride control	0.00088 ± 6.831^b^

a values differ significantly from normal control group.

b values differ significantly from BPH control group.

#### 1.2.2 Determination of prostate specific antigen in blood plasma of experimental animals

Prostate specific antigen (PSA) is found in blood serum when it is increased due to the presence of prostate diseases. The disruption of the epithelium leads to diffusion of PSA into the blood (Table 4).

**Table 4:** Prostate specific antigen (PSA) data in *Wistar* rats treated with *Tetracarpidium conophorum* and induced with benign prostatic hyperplasia.

**Parameters**	**PSA** (ng/ml)
Normal control	0.14 ± 0.018^b^
BPH control	0.3325 ± 0.004^a^
100 mg/kg *Tetracarpidium conophorum*	0.202 ± 0.012^a^^,^^b^
200 mg/kg *Tetracarpidium conophorum*	0.187 ± 0.002^a^^,^^b^
400 mg/kg *Tetracarpidium conophorum*	0.153 ± 0.005^b^
Finasteride control	0.119 ± 0.013^b^

Values are expressed as mean ± SEM (standard error of mean).

a Values differ significantly from normal control.

b Values differ significantly from BPH control.

## 2 Experimental design, materials and methods

Fresh *Tetracarpidium conophorum* were collected within Sango-Ota, Ogun State, Nigeria and was identified at the Botany Department of Covenant University, Ota, Ogun State, Nigeria. The leaves were picked from the branch, air dried and ground to powdered form before use. The pulverized leaves were weighed (600 g), soaked for 3 days in 80% ethanol, sieved and extracted (32 g) using a rotary evaporator.

### 2.1 Phytochemical screening

Qualitative and quantitative phytochemical screening was carried out according to the method described by Tiwari [5] and Varadharajan [6] respectively.

### 2.2 Animals

A total of thirty male Wistar rats (Rattus norvegicus) weighing 170 g to 230 g were purchased from the Federal University of Agriculture, Abeokuta, Nigeria. The rats were housed in the Animal house of Covenant University at room temperature and alternating light cycle 12 h light and dark cycle. All experimental procedures were carried out in compliance with Guidelines for the Care and Use of Laboratory Animals prescribed and approved by Covenant University Health Research Ethics Committee. After one week of acclimatization, the rats were randomly divided into six (6) groups of five (5) rats each.

**Animal treatment:**

**Normal control group**: subcutaneous injection of olive oil.

**BPH control group**: no treatment. 3 mg/kg body weight TP (BPH control group).

**100 mg/kg** ***Tetracarpidium conophorum*** **group**: 100 mg/kg body weight Tetracarpidium conophorumorally and 3 mg/kg body weight TP.

**200 mg/kg** ***Tetracarpidium conophorum*** **group**: 200 mg/kg body weight Tetracarpidium conophorumorally and 3 mg/kg body weight TP.

**400 mg/kg** ***Tetracarpidium conophorum*** **group**: 400 mg/kg body weight Tetracarpidium conophorumorally and 3 mg/kg body weight TP.

**Finasteride control**: 5 mg/kg body weight finasteride and 3 mg/kg body weight TP.

Finasteride was used as the standard drug against BPH and as a positive control. The subcutaneous injection of TP, finasteride and oral administration of the extracts by oral gavage were done daily concurrently (Shin [4]). The rats had unlimited access to food and water throughout the phase of the experiment which lasted for 4 weeks. The body weight was measured weekly. The application volumes were 5 mg/ml/kg for oral administration of finasteride, 100 mg/ml/kg, 200 mg/ml/kg, 400 mg/ml/kg of Laportea aestuans at varying doses and 3 mg/ml/kg for subcutaneous injection of testosterone propionate. After the final treatment, the rats were fasted overnight and anesthetized using diethylether.

### 2.3 Collection of blood and prostate samples

Blood was collected by cardiac puncture into plain bottles, spun for 15 minutes at 3500 rpm to obtain the serum (Iweala and Ogidigo [2], [3]) and then stored at -4C for further analysis.

**Relative Prostate Weight**: prostate weight to body weight ratio was calculated by dividing prostate weight with that of animal body weight (Bhavin [1]).

#### 2.3.1 Statistical analysis

Statistical analysis was carried out using GraphPad Prism (version 7.04 for Microsoft Windows 10) significance by one way analysis of variance (ANOVA), then Fischer׳s Test for post hoc comparison. Data were expressed as mean ± SEM (standard error of mean) of five replicates. Microsoft Excel 2013 was also used in plotting standard curves. Values of *p* < 0.05 were considered statistically significant.
