# Supplementary material for: Prostate specific antigen and relative prostate weight data on effect of Tetracarpidium conophorum leaf extract on testosterone-induced benign prostatic hyperplasia
Source: Data Brief. 2018 Jul 4;20:639–43. doi: 10.1016/j.dib.2018.06.104 (PMC6127981; doi:10.1016/j.dib.2018.06.104)
Supplement: Supplementary file 1 — Supplementary material [file mmc1.docx]

**Conflict of interest**

All the authors confirm as no conflict of interest.
